# Trends in Drug Tests among Children: A 22-Year Retrospective Analysis

**DOI:** 10.3390/pathophysiology30020019

**Published:** 2023-05-12

**Authors:** Carolina Ochoa, Phillip C. S. R. Kilgore, Nadejda Korneeva, Eric Clifford, Steven A. Conrad, Marjan Trutschl, Jacquelyn M. Bowers, Thomas Arnold, Urska Cvek

**Affiliations:** 1 School of Medicine, LSU Health Shreveport, Shreveport, LA 71103, USA; 2 Department of Computer Science, LSU Shreveport, Shreveport, LA 71115, USA; 3 Department of Emergency Medicine, LSU Health Shreveport, Shreveport, LA 71103, USA

**Keywords:** urine drug screening, emergency department, children, neonates, database

## Abstract

There are several pathophysiological outcomes associated with substance abuse including metabolic disbalance, neurodegeneration, and disordered redox. Drug use in pregnant women is a topic of great concern due to developmental harm which may occur during gestation and the associated complications in the neonate after delivery. We sought to determine what the trajectory of drug use is like in children aged 0–4 years and mothers of neonates. Urine drug screen (UDS) results were obtained of our target demographic during 1998–2011 and 2012–2019 from LSU Health Sciences Center in Shreveport (LSUHSC-S). Statistical analysis was performed using R software. We observed an increase in cannabinoid-positive UDS results in both Caucasian (CC) and African American (AA) groups between 1998–2011 and 2012–2019 periods. Cocaine-positive UDS results decreased in both cohorts. CC children had higher UDS positive results for opiates, benzodiazepines, and amphetamines, while AA children had a higher percentage for illicit drugs such as cannabinoids and cocaine. Neonate’s mothers had similar UDS trends to that in children during 2012–2019. Overall, while percentage of positive UDS results for both AA and CC 0–4 year old children started to decline for opiate, benzodiazepine, and cocaine during 2012–2019, cannabinoid- and amphetamine (CC)-positive UDS steadily increased. These results suggest a shift in the type of drug use by mothers from opiates, benzodiazepines, and cocaine to cannabinoids and/or amphetamines. We also observed that 18-year-old females who tested positive for opiates, benzodiazepine, or cocaine had higher than average chances of testing positive for cannabinoids later in life.

## 1. Introduction

The pathophysiological consequences of substance abuse such as altered redox and metabolic pathways leading to tissue damage, as well as the development of mental disorders, were extensively reported [1]. There is a growing health crisis among young women and children due to the exposure to drugs of abuse. In women, substance use may affect not only the physical, but also mental health. The dangers of multi-substance misuse are associated with an increased risk of overdose, impaired physical and mental functions, a decline in quality of life, and mortality due to overdose or suicidal ideation [2,3]. Most substances ingested by a pregnant woman are placentally transferred to the fetus, primarily through a simple diffusion [4,5], which could also lead to stillbirth or cause short- and long-lasting effects on a newborn’s development [6]. Additionally, these effects can persist into adulthood, resulting in learning disabilities, cognitive disorders [7,8,9], and chronic health conditions such as cardiovascular disease [10]. The most attention and research were devoted to adverse effects of opioids, cannabinoids, stimulants, alcohol, and tobacco use during pregnancies [11]. A 2009 study indicated that 23% of pregnant women smoke cigarettes, 20% use alcohol in pregnancy, and 7% of pregnant women self-reported misuses of opioids [12]. As a result, newborns can develop the neonatal abstinence syndrome (NAS), which occurs when a neonate with in utero exposure to certain drugs experiences withdrawal symptoms shortly after birth [13]. Between 2004 and 2014, there was an increase in newborns with NAS from 1.5 to 8 per 1000 hospital births. National data from 2014 reported that a neonate is born with neonatal abstinence syndrome every 15 min [14]. Historically, opioids were associated with NAS, but other drugs such as cocaine and benzodiazepines can result in NAS symptoms [15]. Drug use during pregnancy is also related to many social issues, including poor nutrition, little support for mental health, and male-focused societal structures. Decreased social support, difficulty accessing childcare, and poor support for childcare are associated with substance abuse in pregnancy. The consequences of prenatal and perinatal drug exposure are not fully understood but include a known consequence to fetal brain development. More research needs to be carried out to fully understand the effects and how to mitigate them, allowing for a better understanding of how to manage withdrawal symptoms and treatment of mothers for opioid addiction. While the short- and long-term effects of certain substances such as alcohol and cocaine are established, others such as benzodiazepines remain uncertain [15,16].

In our previous study, we identified the trends of urine drug screen (UDS) results for 18–35 years old women that visited Louisiana State University Health Sciences Center in Shreveport (LSUHSC-S) during the 1998–2019 period [17]. We found that African American (AA) females had a higher percentage of positive UDS results for illicit drugs (cannabinoids and cocaine), while Caucasian (CC) females had a higher percentage of positive UDS results for prescription drugs (opiates, benzodiazepines, and amphetamines). This study analyzed the trends of UDS results for neonate’s mothers and 0–4-year-old children who visited LSUHSC-S during the 1998–2011 and 2012–2019 periods. We also investigated whether drugs used by 18-year-old females could be associated with an increased use of this drug or other drugs later in their life, between ages 19 and 35.

## 2. Materials and Methods

### 2.1. Dataset

Urine drug screen (UDS) results for neonate’s mothers and 0–4-year-old children admitted to the Emergency Department (ED) of Louisiana State University Health Sciences Center at Shreveport (LSUHSC-S) were extracted from two medical record systems. We chose to analyze a 0–4 y.o. group of children based on the assumption that, during this period of life, children are not actively involved in drug ingestion (involuntary drug exposure). This contrasts with other patient populations, who may take the drugs voluntarily. The dataset covering 1998–2011 was manually extracted from medical records prior to implementation of an electronic health record (EHR) system. The dataset covering the 2012–2019 period was extracted from the EPIC electronic medical record system (Verona, WI, USA). Data for the children population covered two periods, 1998–2011 and 2012–2019, and included 8188 and 4769 records, respectively. These records contained demographic information including race, age, payment/insurance type, visit date, and UDS results. The 2012–2019 data for children also included information on the reason for the visit, admission type, and drugs administered during the visit. Data for children during 1998–2011 did not have records about the reason for the visit. Most of the records for 0–4 y.o. children tested during 2012–2019 did not have information about the reason for the LSUHSC-S visit (about 91%). Visit types were categorized into nine types: cardiac/pulmonary, drug misuse, gastroenterology, inflammation/allergy, integument, pain-related, psychiatric/neurologic, sickle cell disease, and other. The dataset for neonate’s mothers included 2414 records collected during the 2012–2019 period. Neonates were defined as 0–7-day-old children. In addition to the demographic information, these datasets also contained records about administration of opiate and benzodiazepine during the neonate mother visit. The UDS was performed for amphetamines, barbiturates, benzodiazepines, cannabinoids, cocaine, ecstasy, methadone, and opiates. The types and methods to perform the UDSs were described in [10]. The immunoassays OPI Flex reagent cartridge (Siemens Healthcare Diagnostics, Inc., Newark, DE, USA) in the Siemens Dimension Vista^®^ System (Siemens Healthineers Medical Solutions USA, Inc., Newark, DE, USA) was used to detect the drugs. In our study, we analyzed UDS results for amphetamines, benzodiazepines, cannabinoids, cocaine, and opiates since other drugs had either no or very low positive rates. There are several limitations associated with the UDS. One limitation is regarding the accuracy of the tests and that, in some cases, they may yield false-positive and false-negative results. Another limitation was that these UDS results were not confirmed by other assays; for example, by the GC/MS or another chemical drug-detection assay.

### 2.2. Data Sources and Analysis

The records from the EHR system contained UDS results, socio-demographic information and visit timestamps. The 2012–2019 data from the EPIC electronic medical record system also included the reason for the visit, drugs administered during the visit, and drug administration timestamps. As the data we received directly from electronic medical records contained some inconsistency, we took several steps to preprocess the data. We treated all instances where a UDS result was marked “detected” or “not detected” as synonymous with a positive or negative result. All remaining results were treated as inconclusive. We also appended neonate’s mothers’ opiate and benzodiazepine administration records during their visit. We performed statistical analysis using R software [18]. UDS results were investigated in the African American (AA) and Caucasian (CC) populations since UDS for other populations (American Indians and Alaska Natives, Native Hawaiians and Other Pacific Islanders) comprised less than 3–4% of total records. Opiate and benzodiazepine prescription rates for neonate’s mothers were analyzed with respect to race and year.

## 3. Results

### 3.1. Demographic Characteristics

During 1998–2011, there were 8507 UDS records for 0–4 y.o. children tested at LSUHSC-S (Table 1). This number decreased to 4769 UDS records in 2012–2019. During both periods, most tests were performed for AA (~75%) and CC children (~20%). Similarly, among 2442 UDS neonate’s mothers tested during 2012–2019, 72% were AA and about 25% were CC mothers (Table 1). Since the combined AA and CC tests accounted for more than 93% of the overall UDS in all three datasets, we analyzed demographic and drug-related data for these two populations. During both periods, the annual number of UDSs performed for AA children was higher each year compared to that of CC children. The annual number of UDSs for AA children dropped by almost 1.5-fold from 629 in 1999 to 424 in 2019 but varied between 82 and 186 for CC children. On average, 296 ± 111 AA and CC women annually visited LSUHSC-S during 2012–2019 period to give birth. Most of the records for 0–4 y.o. children tested at LSUHSC-S during 2012–2019 did not have information about the reason for ED visit (about 91%). Among the remaining 9% of records, 0–4 y.o. children visited most often for the psychiatric/neurologic reasons followed by pain-related, inflammation/allergy-related, cardiac/pulmonary, and gastroenterology-related reasons. The majority of the AA children visits were due to psychiatric/neurologic (129 visits) followed by pain-related (52 visits) issues. Most of the CC children visits were due to pain (33 visits) followed by psychiatric/neurologic (31) reasons. The prevalence of AA over CC children visits was observed in all visit categories.

The most common insurance type for children of both races was Medicaid Managed Care (80% for AA patients, 66% for CC patients), followed by Self-Pay (13% for AA patients, 19% for CC patients). Similarly, among neonate’s mothers, the most common insurance type was Medicaid Managed Care (72.5% for AA, 65.3% for CC mothers), followed by Self-Pay (11% for AA patients, 16% for CC mothers).

### 3.2. Drug Screen Results

**Figure 1:**
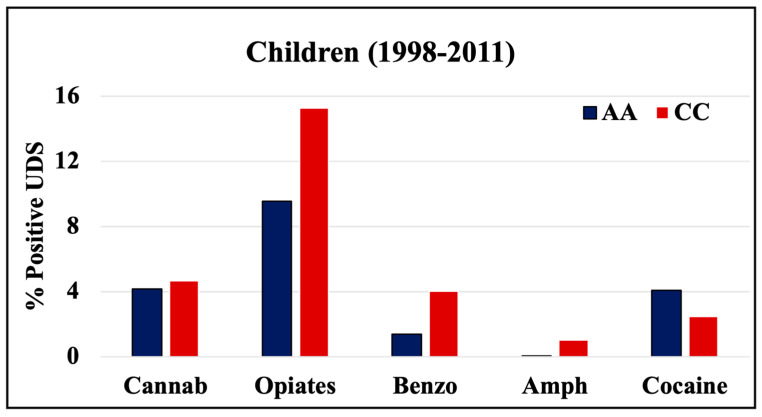
Rates of positive UDS results for children tested at LSUHSC-S during 1998–2011.

**Figure 2:**
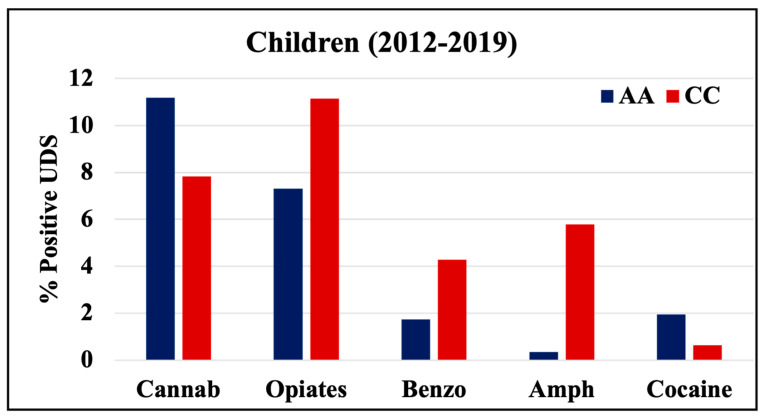
Rates of positive UDS results for children tested at LSUHSC-S during 2012–2019.

**Figure 3:**
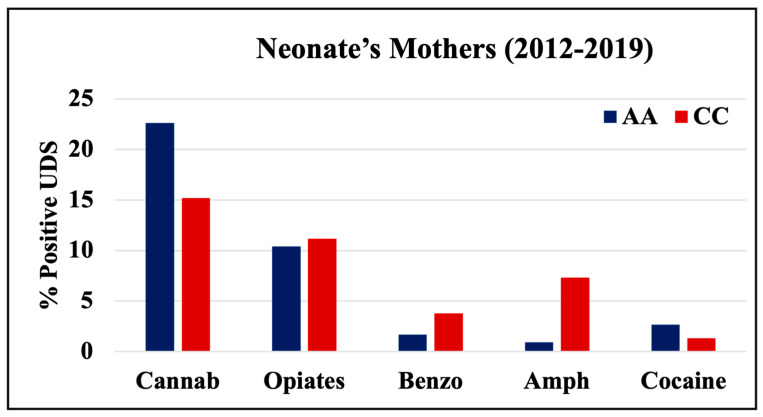
Rates of positive UDS results for neonates’ mothers tested at LSUHSC-S during 2012–2019.

During 1998–2011 and 2012–2019, cannabinoids and opiates were the most commonly positive drugs in the 0–4 y.o. and neonate mothers’ UDS tests (Figure 1, Figure 2 and Figure 3). During the 1998–2011 and 2012–2019 periods, CC children mostly tested positive for opiates, followed by cannabinoids, benzodiazepines, and amphetamines. In 2012–2019, the percentage of positive UDS results for CC children increased in cannabinoids and amphetamine and decreased in opiates and cocaine. During 1998–2011 and 2012–2019, AA children tested positive predominantly for cannabinoids, followed by opiates, and cocaine. In 2012–2019, the percentage of positive UDS results for AA children increased in cannabinoids, and benzodiazepines and decreased in opiates and cocaine. Overall, CC children had a higher percentage of positive UDS results for opiates, benzodiazepines, and amphetamines compared to that in AA children, while AA children had a higher percentage of positive cannabinoids and cocaine results (Figure 1). CC neonate’s mothers tested during 2012–2019 had a higher percentage of positive results for opiates, benzodiazepines, and amphetamines compared to AA mothers, while AA neonate mothers had a higher percentage of positive results for cannabinoids and cocaine (Figure 3).

**Figure 4:**
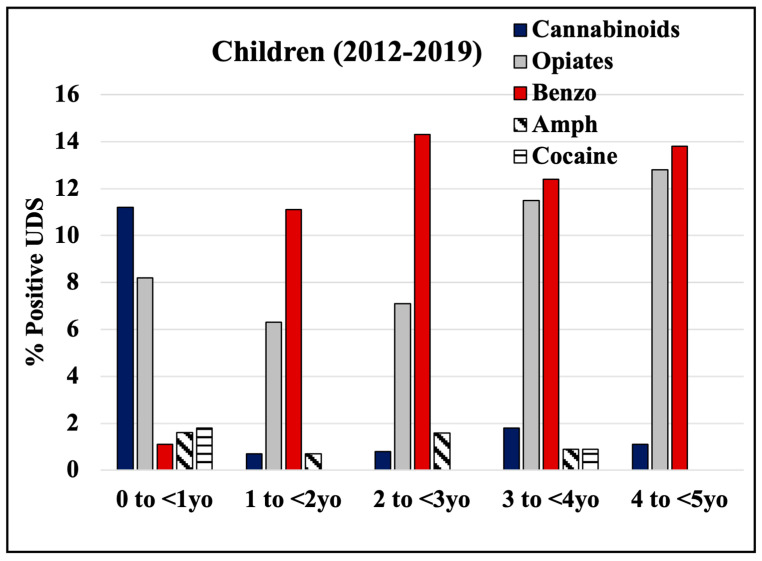
Rates of positive UDS results for children grouped by age group and drug.

An analysis of drug tests in separate age groups of children revealed that the highest positive results were for cannabinoids (11%) in the “0 to younger than 1 y.o.” children group, which switched to benzodiazepines (between 11–14%) in children 1 y.o. and older (Figure 4). The second highest positive results were for opiates: 6–8% in children younger than 3 y.o. that increased to 11–13% in children 3 y.o. and older (Figure 4). These data suggest that mothers switch exposure of children from cannabinoids to benzodiazepines at around 1 year of age.

### 3.3. Trends in Positive UDS Results

#### 3.3.1. Opiates

**Figure 5:**
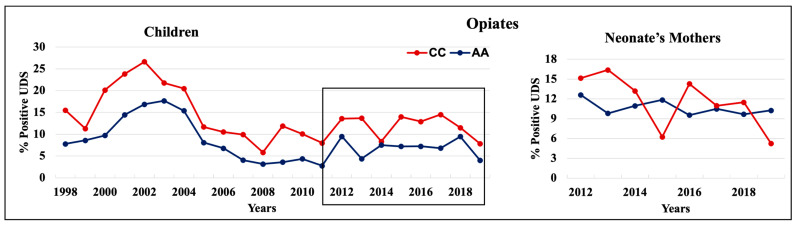
Trends in the rate of positive UDS results for children and neonate’s mothers for opiates.

Each year during 1998–2019, CC 0–4 y.o. children had higher positive opiate UDS rates compared to AA children (Figure 5, left panel). Interestingly, the opioid UDSs in both CC and AA children population spiked up to 27% (CC) and 18% (AA) in 2002–2003 and then declined in 2005, remaining relatively constant at 10–15% (CC) and 3–10% (AA) after that. Similarly, during these periods, CC neonates’ mothers tested positive for opiates more often than AA mothers each year except in 2015 and 2019 (Figure 5, right panel). The opiate positive UDS results also included the data of how many patients received opiates during their ED visit. Thus, we analyzed records for opiate administration among neonate’s mothers. During 2012–2019, the rate of opiate administration during the visits was similar between AA and CC neonate mothers (25–30%) with the exception of 2016 when CC had higher opiate administration rate (40%) compared to AA neonate mothers.

#### 3.3.2. Benzodiazepine

**Figure 6:**
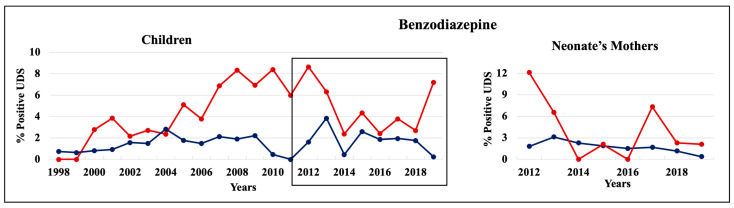
Trends in the rate of positive UDS results for children and neonate’s mothers for benzodiazepine.

Starting from 2000, CC children had higher positive benzodiazepines UDS rate compared to AA children reaching 8% in 2007–2012, which then declined to 4% (Figure 6, left panel). The percentage of positive benzodiazepines UDS among AA children was between 1 and 3.8% during 1998–2019. The rate of positive UDS for benzodiazepines in neonate’s mothers population was similar to that in children (Figure 6, right panel). The benzodiazepine positive UDS results also included the data of how many patients received the drug during their ED visit. Thus, we analyzed benzodiazepine administration records for neonate’s mothers during their visit to the LSUHSC-S in 2012–2019. The rate of benzodiazepine administration was slightly higher in CC neonate’s mothers’ population, decreasing from ~13% in 2012 to under 3% during 2014–2019. In AA neonate’s mothers population, the rate of benzodiazepines administration during their visit was relatively constant, under 3% during the entire 2012–2019 period.

#### 3.3.3. Amphetamine

**Figure 7:**
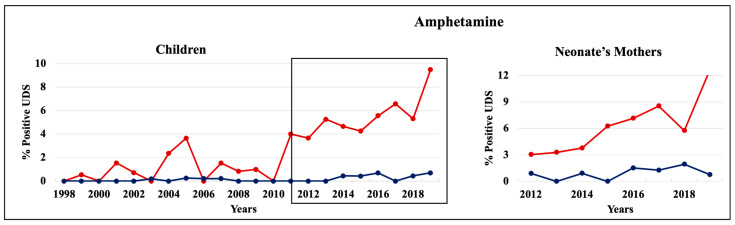
Trends in the rate of positive UDS results for children and neonate’s mothers for amphetamines.

Starting from 2011, the percentage of amphetamine positive UDS results increased dramatically in CC 0–4 y.o. children and neonate mothers populations, spiking to 9.5% in children and to 12% in mothers in 2019 (Figure 7). In contrast, AA children and neonate mothers tested positive for amphetamines in less than 1% and 2%, respectively, during the periods tested.

#### 3.3.4. Cannabinoid

**Figure 8:**
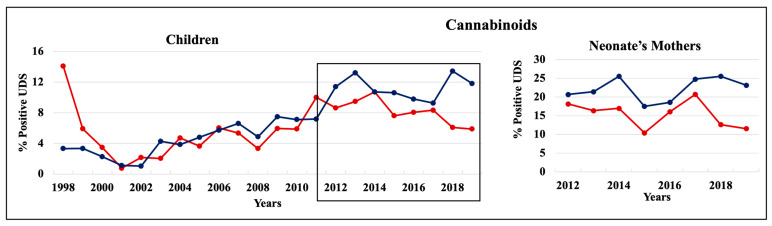
Trends in the rate of positive UDS results for children and neonate’s mothers for canna-binoids.

During 1998–2019, the percentage of cannabinoid-positive UDS results increased in both AA and CC 0–4 y.o. children’s populations, reaching 13% and 11%, respectively (Figure 8, left panel). AA children had a higher cannabinoids rate than CC in most of this period. Similarly, AA neonate’s mothers tested positive for cannabinoids at a higher rate (21–26%) compared to CC (12–21%) mothers (Figure 8, right panel).

#### 3.3.5. Cocaine

**Figure 9:**
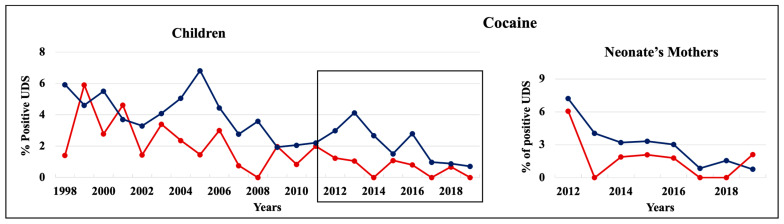
Trends in the rate of positive UDS results for children and neonate’s mothers for cocaine.

During 1998–2019, the percentage of cocaine positive UDS results decreased from around 6% to less than 1% in both AA and CC children’s populations (Figure 9, left panel). AA children had a higher cocaine rate than CC each year except 1999 and 2001. Similarly, among neonate’s mothers, the percentage of cocaine positive UDS results slightly decreased in both AA (from 7% to 0.8%) and CC (from 6% to 2%) populations (Figure 9, right panel). AA neonate’s mothers tested positive for cocaine with higher rates each year during 2012–2019, except in 2019.

#### 3.3.6. Trends in Drug Use among 18 Years Old Females Later in Their Life

To determine whether drugs used by 18 y.o. females could be associated with use of this or other drugs, we investigated the trends in the UDS results among 358 eighteen-year-old females who had subsequent UDS tests performed when they visited LSUHSC-S later in their life, between the ages of 19 and 35 (Table 2). We compared their results to the records of all 18–35 y.o. females who visited LSUHSC-S during 2012–2019 (Table 2, row “All 18–35 y.o. females”). Our analysis revealed that when 18 y.o. females had positive UDS for cannabinoids, they were more likely to test positive for cannabinoids, amphetamine, and cocaine later in life compared to a female from the 18–35 y.o. population (Table 2, row “Cannabinoid” vs. “All 18–35 y.o. females”). When 18 y.o. females tested positive for opiates, they were more likely to test positive for cannabinoids, opiates, benzodiazepines, and amphetamine later in life (Table 2, row “Opiate”). Benzodiazepine positive UDS results at age 18 would likely indicate higher positive rates for cannabinoids, benzodiazepine, and amphetamine later in life (Table 2, row “Benzodiazepine”). When 18 y.o. females tested positive for amphetamine, they were more likely to test positive for cannabinoids, benzodiazepines, amphetamine, and cocaine later in life compared to a female from the 18–35 years old population (Table 2, row “Amphetamine”). Cocaine-positive UDS results at 18 would likely indicate higher positive rates for cannabinoids, amphetamine, and cocaine later in life (Table 2, row “Cocaine”).

## 4. Discussion

In this study, we investigated the trends in UDS results of 0–4 years old children who visited LSUHSC-S during two periods, 1998–2011 and 2012–2019. In addition, we analyzed the trends of UDS results for neonate’s mothers who gave birth at LSUHSC-S during 2012–2019. During both periods, the majority of tests were performed for AA (~75%) and CC children (~20%), similar to the population of neonate mothers tested during 2012–2019: 72% and ~25% for AA and CC mothers, respectively. Interestingly, these demographic profiles differed from the population of young females of childbirth age tested in the hospital during 2012–2019: 63% AA and 35% CC [17], which is close to the population distribution of Shreveport: 57% AA and 31% CC [19]. These data suggest that females who gave birth at the LSUHSC-S preferred to bring their children to LSUHSC-S in case of emergency.

In our study, we observed that during both periods, 1998–2011 and 2012–2019, CC children had a higher percentage of positive UDS results for prescription medications such as opiates, benzodiazepines, and amphetamines compared to that in AA children. AA children had higher percent of positive UDS results for illicit drugs, such as cannabinoids and cocaine. Not surprisingly, neonate’s mothers tested during 2012–2019 had a similar UDS trend. In our recent study of UDS results for 18–35 y.o. females, we observed a similar trend [17]. These results suggest that mother’s drugs use habits influence what drugs their children are involuntarily exposed to during early life.

We discovered that opiates were one of the most often detected substances in the UDS tests in AA and CC children: 10% of 0–4 years old children tested opiate-positive during 1998–2019, with CC children having higher records over AA children. The percentage of opiate-positive UDS varied between 8% (younger than 1 year old) and 13% (4 years old). Similarly, about 10% of neonate mothers in both populations, AA and CC, tested positive for opiate, with CC mothers having slightly higher opiate positive UDS records. At least one in ten neonates born at LSUHSC-S during 2012–2019 were exposed to opiates in utero. Opioids have a well-established association with NAS and may result in adverse outcomes such as abruptio placentae, SIDS (sudden infant death syndrome), decreased in utero growth leading to low birthweight and smaller head circumference, increased risk of in utero death, and infant mortality [20,21] as well as congenital defects including oral clefts, cardiac malformations, and neural tube defects [21,22,23]. This eventually interferes with neural networks involved with motor and behavior regulation [24,25], all which may later affect scholastic performance.

The opiate administration rate during the visit to the hospital was similar between CC and AA neonate mothers during 2012–2019. This result suggests that while in the hospital CC and AA these mothers have equal access to opiate treatment, outside of the hospital CC population probably has an easier access to opiates. This agrees with our previous study in which we observed a higher percentage of opiate-positive UDS in CC females compared to that in AA population [17]. Opioid exposure early in life may lead to opioid dependence. In our study, we observed that among 18 y.o. females who tested positive for opiates, 43% would be having opiate positive UDS results later in life, at age 19–35 years old, which was higher than 18% of 18–35 years old female population.

We discovered that benzodiazepine UDS positivity was consistently higher in the CC populations of 0–4 y.o. children and neonate’s mothers. The rate of benzodiazepine administration during the visit also was slightly higher in the CC neonate’s mothers’ population compared to AA population. In our previous study, we observed a similar trend among young females of childbearing ages. CC females had up to 5-fold higher percentage of benzodiazepine positive UDS records compared to AA females during 1998–2019 [17]. These results indicate that both the in-hospital and outside of hospital CC population has higher rate of benzodiazepine use compared to AA population.

While the percentage of benzodiazepine-positive UDS was about 1% in children younger than 1 year old, it drastically increased to 14% in the older children population. Benzodiazepines are generally prescribed to treat anxiety or seizures in children as young as 6 months old. Our records did not provide information whether benzodiazepine-positive UDS results among children were due to a child’s prescription. The effects of in utero benzodiazepine exposure are associated with lower birth weight, preterm birth, or abnormal development [26,27,28,29]. Due to the depressant nature of benzodiazepines and their ease to cross the placenta, hypoventilation and hypertonicity were noted in neonates with exposure late in gestation and has been called “floppy infant syndrome” [30]. The long-term effects of benzodiazepines include impaired motor skills, internalizing problems, and increased attention-deficit/hyperactivity disorder traits [15,31,32,33]. Benzodiazepine misuse was reported to be associated with misuse of other substances [34] and with higher levels of polysubstance abuse [34,35]. Indeed, in our study, we observed that benzodiazepine positive UDS results in 18-year-old females associated with more frequent positive results for cannabinoid and amphetamine later in life, suggesting co-use in this cohort.

We observed that both AA and CC children had an increased percentage of positive cannabinoid UDS results during 2012–2019 vs. 1998–2011. Compared to other drugs, cannabinoid-positive UDSs were the highest among neonate’s mothers and children younger than 1 years old during 2011–2019, suggesting that children were exposed to cannabinoids in utero and/or during their first year of life. Cannabinoid exposure early in life may contribute to several complications in physical, behavioral, and cognitive development [36,37,38,39]. Several studies found that maternal marijuana use can alter the expression of brain receptors and the neural connectivity of brain regions that regulate emotions, potentially leading to cognitive and behavioral issues for the neonate as they grow [40,41] as well as an increased risk of developing depression, behavioral issues, and substance abuse disorders in teenage years [42,43,44].

We discovered that amphetamine UDS records were not just higher in the CC 0–4 years old children population during 1998–2019, but starting from 2012, it tripled to almost 10% in the CC population. The increase in amphetamine UDS records among CC children correlated with a similar increase in the neonate’s mothers’ population that reached almost 13% by 2019. These results suggest that starting from 2012, increasing number of CC neonates were exposed to amphetamines in utero. In contrast, in the AA 0–4 y.o. children and neonate’s mothers, percentage of amphetamine-positive UDS results stayed low, under 1% and 2%, respectively, over the periods tested. Our finding of a sharp increase in amphetamine-positive UDS records among CC children and neonate’s mothers correlated with almost 3-fold increase in amphetamine positive UDSs among 18–35 y.o. females tested at LSUHSC-S during the same time period [17]. Moreover, in our current study, we found that amphetamine positive UDS results in 18 y.o. females associated with more frequent positive results for cannabinoid, benzodiazepine, cocaine, and amphetamine later in life, suggesting co-use in this cohort. Indeed, use of any of cannabinoids, opiates, benzodiazepine, or cocaine by 18 y.o. female correlated with increased chances to test positive for amphetamine later in life. Amphetamine use during pregnancy may be associated with several adverse effects associated with childbirth and development. Amphetamines are classified as stimulants and sympathomimetics which can cross and accumulate in the placenta [45,46]. Maternal amphetamine use was associated with several complications such as placental hemorrhage, preterm labor, and in utero growth restriction leading to lower birth weight, head circumference, and body length [45,46] as well as cognitive and motor impairments [47,48,49].

We found that the percentage of cocaine-positive UDS records decreased in both AA and CC 0–4 y.o. children populations tested during 1998–2019 period, with AA children having a higher percentage almost every year. We observed a similar decline in cocaine UDS results among neonate’s mothers with AA mothers having higher percentage almost every year except 2019. Recently, it was reported that the availability of cocaine was in decline (in part) due to the increased regulation of the precursor chemical potassium permanganate [50]. Thus, it may be the case that lack of general availability drove this decrease. We found that among 0–4 y.o. cohort, children younger than 1 years old tested positive for cocaine more often than other age groups, comprising 2%. These results suggest that almost 2% of newborns were exposed to cocaine in utero or during the first year of their life. Cocaine use during pregnancy may be associated with several adverse effects associated with childbirth and development. Similar to amphetamines, cocaine is a stimulant with vasoconstrictive properties that easily crosses the placenta and increases the amount of dopamine, serotonin, and norepinephrine [51]. However, unlike them, cocaine in a known teratogen and was linked to numerous structural abnormalities such as cardiac and skeletal defects [51,52,53]. Disturbed sleep patterns, hypertonia, and impaired suckling reflex were also observed with cocaine exposure, just as with methamphetamine exposure [52]. Therefore, in utero complications of prenatal cocaine use are similar to those of prenatal amphetamine use, with a stronger association compared to methamphetamines.

## 5. Conclusions

Overall, while the percentage of positive UDS results for both AA and CC 0–4 years old children started to decline for opiate, benzodiazepine, and cocaine, at the same time, cannabinoid-positive UDS steadily increased starting from 2005. These results suggest a shift in the type of drug used by mothers from opiates, benzodiazepines, and cocaine, to cannabinoids. In our study, we observed that 18-year-old females who tested positive for opiates, benzodiazepine, or cocaine had higher than average chances to test positive for cannabinoids later in life. We also observed a sharp increase in amphetamine-positive UDS results in the CC 0–4 years old population starting from 2011.

## Figures and Tables

**Table 1:** Racial Demographics of UDS Records by Timeframe.

	0–4-Year-Old Children		Neonates’ Mothers
Self-Identified Race	1998–2011	2012–2019	2012–2019
Black or African American	6453	3530	1758
White or Caucasian	1735	932	606
American Indian/Alaska Native	10	5	4
Asian	28	9	2
Native Hawaiian and Other Pacific Islander	242	9	4
Other	39	3	51
Unknown	0	155	17
Total	8507	4769	2442

**Table 2:** Percentage of positive UDS results in those 19-35 years old females who tested positive for a certain drug when they were 18 years old.

	Drug at 19	Cannabinoid	Opiate	Benzodiazepine	Amphetamine	Cocaine
Drug at 18						
Cannabinoid		86	11	7	35	14
Opiate		39	43	19	16	0
Benzodiazepine		67	12	21	67	9
Amphetamine		76	18	26	71	45
Cocaine		65	0	0	50	30
All 18-35 y.o. females		31	18	7	8	5

## Data Availability

The data that support the findings of this study are available from Ochsner LSU Health, but restrictions apply to the availability of these data, which were used under license for the current study, and so, are not publicly available. Data are, however, available from the authors upon reasonable request and with permission of LSU Health Shreveport and Ochsner LSU Health.
